# The Effects of Freezing on Faecal Microbiota as Determined Using MiSeq Sequencing and Culture-Based Investigations

**DOI:** 10.1371/journal.pone.0119355

**Published:** 2015-03-06

**Authors:** Fiona Fouhy, Jennifer Deane, Mary C. Rea, Órla O’Sullivan, R. Paul Ross, Grace O’Callaghan, Barry J. Plant, Catherine Stanton

**Affiliations:** 1 Teagasc Food Research Centre, Moorepark, Fermoy, County Cork, Ireland; 2 HRB Clinical Research Facility, University College Cork, Cork, Ireland; 3 School of Microbiology, University College Cork, Cork, Ireland; 4 Alimentary Pharmabiotic Centre, Cork, Ireland; 5 College of Science, Engineering and Food Science (SEFS), University College Cork, Cork, Ireland; 6 Cork Adult Cystic Fibrosis Centre, University College Cork, Cork University Hospital, Wilton, Cork, Ireland; University of Florida, UNITED STATES

## Abstract

**Background:**

High-throughput sequencing has enabled detailed insights into complex microbial environments, including the human gut microbiota. The accuracy of the sequencing data however, is reliant upon appropriate storage of the samples prior to DNA extraction. The aim of this study was to conduct the first MiSeq sequencing investigation into the effects of faecal storage on the microbiota, compared to fresh samples. Culture-based analysis was also completed.

**Methods:**

Seven faecal samples were collected from healthy adults. Samples were separated into fresh (DNA extracted immediately), snap frozen on dry ice and frozen for 7 days at -80°C prior to DNA extraction or samples frozen at -80°C for 7 days before DNA extraction. Sequencing was completed on the Illumina MiSeq platform. Culturing of total aerobes, anaerobes and bifidobacteria was also completed.

**Results:**

No significant differences at phylum or family levels between the treatment groups occurred. At genus level only *Faecalibacterium* and *Leuconostoc* were significantly different in the fresh samples compared to the snap frozen group (p = 0.0298; p = 0.0330 respectively). Diversity analysis indicated that samples clustered based on the individual donor, rather than by storage group. No significant differences occurred in the culture-based analysis between the fresh, snap or -80°C frozen samples.

**Conclusions:**

Using the MiSeq platform coupled with culture-based analysis, this study highlighted that limited significant changes in microbiota occur following rapid freezing of faecal samples prior to DNA extraction. Thus, rapid freezing of samples prior to DNA extraction and culturing, preserves the integrity of the microbiota.

## Introduction

The human gut microbiota is a complex ecosystem, comprised of thousands of bacterial species which is increasingly being investigated for its role in health and disease [1]. Such complexity and diversity poses considerable challenges to researchers as to how best to investigate such an environment accurately and completely. In the past, studies relied heavily on culture-based approaches, known now to capture only 10–20% of the actual microbiota present [2]. However, as molecular technologies advanced, studies increasingly applied molecular approaches such as PCR, denaturing gradient gel electrophoresis or temperature gradient gel electrophoresis to determine the gut microbiota with greater accuracy than culturing alone [3]. Over the past 2 decades, with our enhanced understanding of the human gut microbiota, researchers have investigated the contribution of gut microbes to diseases such as diabetes, obesity, inflammatory bowel disease and certain cancers [4–6]. These investigations have been enabled through the advent of next generation sequencing technologies including Roche 454-pyrosequencing, Illumina MiSeq and PacBio [7].

With decreasing costs and increasing speed, high-throughput sequencing approaches are being applied more frequently to investigate human gut microbiota. The majority of sequencing studies sequence the hyper variable regions of the 16S ribosomal RNA (rRNA) bacterial gene. This enables the sequencing of all bacteria, without requiring prior knowledge of which populations are present, while also providing insights into populations present at low abundances that could be missed by culturing alone. The results from high-throughput sequencing can however be biased by numerous factors. Studies have shown that the DNA extraction procedure, primers and sequencing platform used, can impact on the accuracy of the results achieved [8–10]. In the majority of gut microbiota studies, faecal samples are used owing to the non-invasive nature of collecting such samples. Recently, studies have highlighted that the storage conditions of the sample prior to DNA extraction can alter the microbiota detected, especially in cases where prolonged room temperature exposure occurs [11]. However, at present limited studies have addressed a critical comparison of microbiota composition in fresh faecal samples to samples snap frozen on dry ice, to samples frozen immediately at -80°C, using both culturing methods and sequencing on the MiSeq platform. In fact, there is a considerable paucity of studies investigating the ability to culture from frozen faecal samples. This is critical information as increasingly research collaborations between institutions are occurring, resulting in a necessity to collect, store and ship samples in an appropriate manner to enable accurate subsequent culture-based analysis. While studies using 454-pyrosequencing have been completed on this topic, to date we are unaware of any study using MiSeq sequencing to investigate the effects of such storage conditions on faecal microbiota. Given the increased sequencing depth achieved through MiSeq sequencing compared to 454-pyrosequencing, subtle changes in microbiota that may not be detected using 454-pyrosequencing, may be captured using the MiSeq sequencing platform. Thus, the aim of this study was to compare the faecal microbiota of 7 healthy adults using culturing and MiSeq sequencing approaches and to determine the effects of different storage conditions on the faecal microbiota detected. Such results will inform future studies using faecal samples and MiSeq sequencing on the most appropriate way to store samples prior to DNA extraction, when extraction from fresh samples is not appropriate.

## Materials and Methods

### Sample collection and experimental design

Fresh faecal samples were collected from 7 individuals (5 females). Participants were healthy adults, free from gastrointestinal conditions, with no antibiotic exposure in the 28 days prior to sample collection. Participants provided written informed consent. Ethical approval was received from the Clinical Research Ethics Committee of the Cork Teaching Hospitals, Cork, Ireland. Fresh samples were collected and aliquoted within 4 hours of defecation. Each sample was homogenised and 250mg aliquots from each faecal sample was added to 3 separate cryovials (Sarstedt, Wexford, Ireland) containing zirconia/silica bead mix (Stratech Scientific, UK). One aliquot per individual was immersed in dry ice for 4 minutes until completely frozen. These ‘snap’ frozen samples were then stored at -80°C for 7 days before DNA extractions and culturing were completed. The ‘frozen’ samples were immediately frozen at -80°C for 7 days following collection and aliquoting. The ‘fresh’ samples were processed within 4 hours of collection, and were stored at 4°C during this brief period between collection and DNA extraction. In the case of culture-based analysis, 1g was taken from each homogenised faecal sample and stored under the 3 storage conditions detailed above, before being used for culturing.

### Culture-based analysis

The effects of sample storage conditions on microbial populations were determined using culture-based approaches targeting total aerobes, total anaerobes and total bifidobacteria. One gram of fresh, snap or -80°C frozen faecal material per individual was taken and serially diluted in maximum recovery diluent (MRD) (Oxoid Ltd, Basingstoke, Hampshire, UK). Dilutions were then plated in triplicate on the respective media. Enumeration of total aerobes was performed by plating samples from dilutions 10^−4^–10^−7^ onto Brain Heart Infusion (BHI) agar (Merck, Darmstadt, Germany) supplemented with 50 units of Nystatin (Sigma Aldrich, Dublin, Ireland) and incubated for 5 days at 37°C, aerobically. Enumeration of total anaerobes was determined by plating samples from dilutions 10^−4^–10^−7^ on Wilkins Chalgren Agar (WCA) (Fluka analytical) supplemented with 50 units of Nystatin and 7.5% defibrinated horse blood (TCS Biosciences, Buckingham, UK) and incubated for five days at 37°C anaerobically. Enumeration of bifidobacteria was assessed by plating samples from dilutions 10^−1^–10^−5^ for fresh samples, from 10^−4^–10^−7^ for -80°C frozen and snap frozen samples onto de Man Rogosa Sharpe (MRS, Difco Laboratories, Detroit, MI, USA) agar supplemented with 0.05% (wt/vol) L-cysteine hydrochloride (Sigma Aldrich), 100 μg/ml Mupirocin (Fluka) [12] and 50 units of Nystatin. These plates were then incubated at 37°C for 72 hours under anaerobic conditions (Anaerocult A gas packs; Merck). Following incubation, enumeration using plate counts was performed for all three groups.

### DNA extraction

Total bacterial metagenomic DNA was extracted using a modified protocol which combined the repeat bead beating method [13] with the QIAmp Fast DNA Stool Mini kit (Qiagen, UK). DNA from the fresh sample was extracted within 4 hours of collection, while DNA from the snap and -80°C frozen samples was extracted after 7 days at -80°C. Briefly, 1ml of lysis buffer (500mM NaCl, 50mM Tris-HCl pH8.0, 50mM EDTA and 4% sodium dodecyl sulphate) was added to the bead beating tubes containing the faecal sample. Samples were homogenised for 3 mins at max speed using the Mini Beadbeater (BioSpec). Samples were incubated at 70°C for 15mins to lyse the cells. Following centrifugation the supernatant was removed and the bead beating steps repeated. Following pooling of the supernatant, samples were treated with 10M ammonium acetate (Sigma Aldrich, Ireland) and then the DNA was pelleted and washed with 70% ethanol. The DNA was then RNAse and proteinase K treated. Finally the DNA was washed using buffers AW1 and AW2 (QIAmp Fast DNA Stool Mini kit; Qiagen, UK) and eluted in 200 μl of ATE buffer. DNA was quantified using the Nanodrop 1000 (Thermo Scientific, Ireland).

### 16S rRNA amplification and MiSeq sequencing

The V3-V4 variable region of the 16S rRNA gene was amplified from 21 faecal DNA extracts using the 16S metagenomic sequencing library protocol (Illumina). Two PCR reactions were completed on the template DNA. Initially the DNA was amplified with primers specific to the V3-V4 region of the 16S rRNA gene which also incorporates the Illumina overhang adaptor (Forward primer 5’ TCGTCGGCAGCGTCAGATGTGTATAAGAGACAGCCTACGGGNGGCWGCAG; reverse primer 5’ GTCTCGTGGGCTCGGAGATGTGTATAAGAGACAGGACTACHVGGGTATCTAATCC). Each PCR reaction contained DNA template (~10–12ng), 5 μl forward primer (1 μM), 5 μl reverse primer (1 μM), 12.5 μl 2X Kapa HiFi Hotstart ready mix (Anachem, Dublin, Ireland), PCR grade water to a final volume of 25μl. PCR amplification was carried out as follows: heated lid 110°, 95°C x 3mins, 25 cycles of 95°C x 30s, 55°C x 30s, 72°C x 30s, then 72°C x 5mins and held at 4°C. PCR products were visualised using gel electrophoresis (1X TAE buffer, 1.5% agarose, 100V). Successful PCR products were cleaned using AMPure XP magnetic bead based purification (Labplan, Dublin, Ireland). A second PCR reaction was completed on the purified DNA (5μl) to index each of the samples, allowing samples to be pooled for sequencing on the one flow cell and subsequently demultiplexed for analysis. Two indexing primers (Illumina Nextera XT indexing primers, Illumina, Sweden) were used per sample. Each PCR reaction contained 5μl index 1 primer (N7xx), 5μl index 2 primer (S5xx), 25μl 2x Kapa HiFi Hot Start Ready mix, 10μl PCR grade water. PCRs were completed as described above, but only 8 amplification cycles were completed instead of 25. PCR products were visualised using gel electrophoresis and subsequently cleaned (as described above). Samples were quantified using the Qubit (Bio-Sciences, Dublin, Ireland), along with the broad range DNA quantification assay kit (BioSciences) and samples were then pooled in an equimolar fashion. The pooled sample was run on the Agilent Bioanalyser for quality analysis prior to sequencing. The sample pool (4nM) was denatured with 0.2N NaOH, then diluted to 4pM and combined with 10% (v/v) denatured 4pM PhiX, prepared following Illumina guidelines. Samples were sequenced on the MiSeq sequencing platform in the Teagasc sequencing facility, using a 2 x 300 cycle V3 kit, following standard Illumina sequencing protocols.

### Bioinformatics and statistical analysis

Three hundred base pair paired-end reads were assembled using FLASH (FLASH: fast length adjustment of short reads to improve genome assemblies). Further processing of paired-end reads including quality filtering based on a quality score of > 25 and removal of mismatched barcodes and sequences below length thresholds was completed using QIIME. Denoising, chimera detection and clustering into operational taxonomic units (OTUs) (97% identity) were performed using USEARCH v7 (64-bit) [14]. OTUs were aligned using PyNAST (PyNAST: python nearest alignment space termination; a flexible tool for aligning sequences to a template alignment) and taxonomy was assigned using BLAST against the SILVA SSURef database release 111. Alpha and beta diversities were generated in QIIME [15] and calculated based on weighted and unweighted Unifrac distance matrices. Principal coordinate analysis (PCoA) plots were visualised using EMPeror v0.9.3-dev.

To determine if statistically significant differences occurred in microbial populations between the 3 storage groups, non-parametric Kruskall-Wallis analysis was completed using Minitab 15 statistical software package. Statistical significance was accepted as p<0.05, adjusted for ties. Microbiological enumeration data was log10 transformed prior to statistical analysis using GraphPad Prism 6 and RM one-way ANOVA testing with Geisser-Greenhouse correction.

## Results

### Culture-based analysis

**Fig 1:**
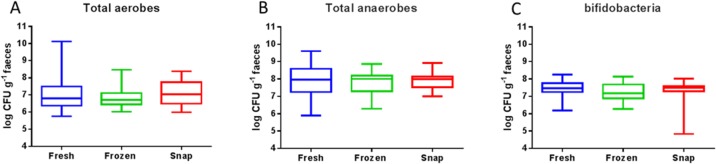
Microbiological enumeration of total aerobes (A), total anaerobes (B) and bifidobacteria (C) in log CFUg-1 faeces from fresh (blue), -80°C frozen (green) and snap samples (red). RM one-way ANOVA statistical testing showed no significant difference between storage conditions fresh, frozen at -80°C or snap frozen for the three groups tested.

To assess the effects of different storage conditions on the ability to culture anaerobes, aerobes and bifidobacteria, culture-based analysis was performed. Following incubation, enumeration using plate counts was performed for all three groups. Total aerobic populations ranged from 5.75–10.11 log colony forming units (CFU) g^−1^ (mean = 7.06 log CFU g^−1^) from fresh samples, 6.02–8.47 log CFU g^−1^ (mean = 6.89 log CFU g^−1^) for -80°C frozen samples and 5.98–8.37 log CFU g^−1^ (mean = 7.11 log CFU g^−1^) for snap frozen samples. No significant differences between enumerated aerobic populations were found in the 3 different storage groups (p = 0.686; Fig. 1A). Total anaerobic populations ranged from 5.88–9.60 log CFU g^−1^ (mean = 7.99 log CFU g^−1^) for fresh faecal samples, 6.28–8.86 log CFU g^−1^ (mean = 7.78 log CFU g^−1^) for -80°C frozen samples and 7.00–8.92 log CFU g^−1^ (mean = 7.89 log CFU g^−1^) for samples plated from snap frozen faeces. No significant difference in numbers of total anaerobes occurred depending on storage conditions (p = 0.350; Fig. 1B).

Bifidobacteria counts ranged from 6.18–8.24 log CFU g^−1^ (mean = 7.56 log CFU g^−1^) for fresh faeces, 6.27–8.12 log CFU g^−1^ (mean = 7.22 log CFU g^−1^) for -80°C frozen samples and 4.82–8.02 log CFU g^−1^ (mean = 7.30 log CFU g^−1^) for the snap frozen samples. No significant difference existed between bifidobacteria enumerated from the samples at different storage conditions (p = 0.334; Fig. 1C).

### MiSeq analysis of faecal microbiota following different storage conditions

Following the extraction of DNA from the fresh, -80°C frozen and snap frozen faecal samples, MiSeq sequencing was used to determine the effects of storage on faecal microbiota. Samples were sequenced using the Illumina MiSeq platform, resulting in 13.7 million reads.

**Fig 2:**
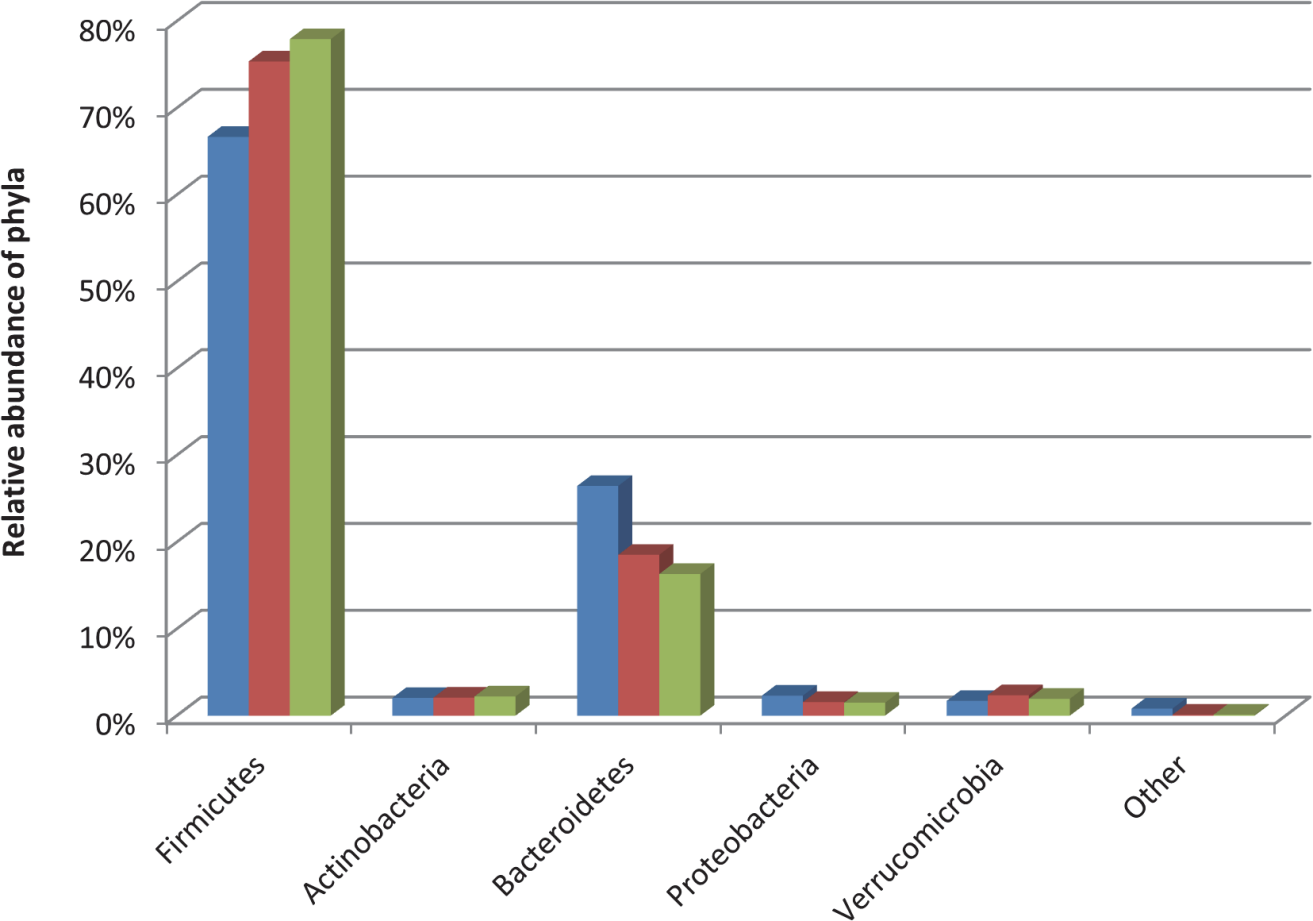
Relative abundances of bacterial phyla in the fresh (blue), snap (red) and -80°C frozen samples (green). Other contains phyla present at < 1% of assignable sequences at phylum level.

At phylum level the fresh, snap and -80°C frozen samples shared common phyla. The most prevalent phyla in all samples were the *Firmicutes* and *Bacteroidetes* with *Actinobacteria*, *Fusobacteria* and *Cyanobacteria* contributing smaller proportions of the sequencing reads. No significant differences between the levels of *Bacteroidetes* (p = 0.428) or *Actinobacteria* (p = 0.901) occurred (Fig. 2). While there was an increase in the proportion of *Firmicutes* present in the -80°C frozen (78%) and snap frozen samples (75%) compared to the fresh samples (67%), this was not significant (p = 0.205). The most prevalent families detected in all 3 treatment groups included *Ruminococcaceae*, *Lachnospiraceae*, and *Bacteroidaceae*. However, again storage conditions appeared to have no significant effects, as the proportions of any of the families present in the frozen and snap samples were not significantly different to the fresh samples (*Bifidobacteriaceae* p = 0.809, *Bacteroidaceae* p = 0.667, *Lactobacillaceae* p = 0.912, *Ruminococcaceae* p = 0.754 and *Lachnospiraceae* p = 0.511). Additionally, populations that only contributed a minor proportion to the overall gut microbiota i.e. <1% of total reads e.g. *Verrucomicrobiaceae*, *Pseudomonadacea*e, were also equally detected in all 3 groups (p = 0.996 and p = 0.880 respectively).

**Fig 3:**
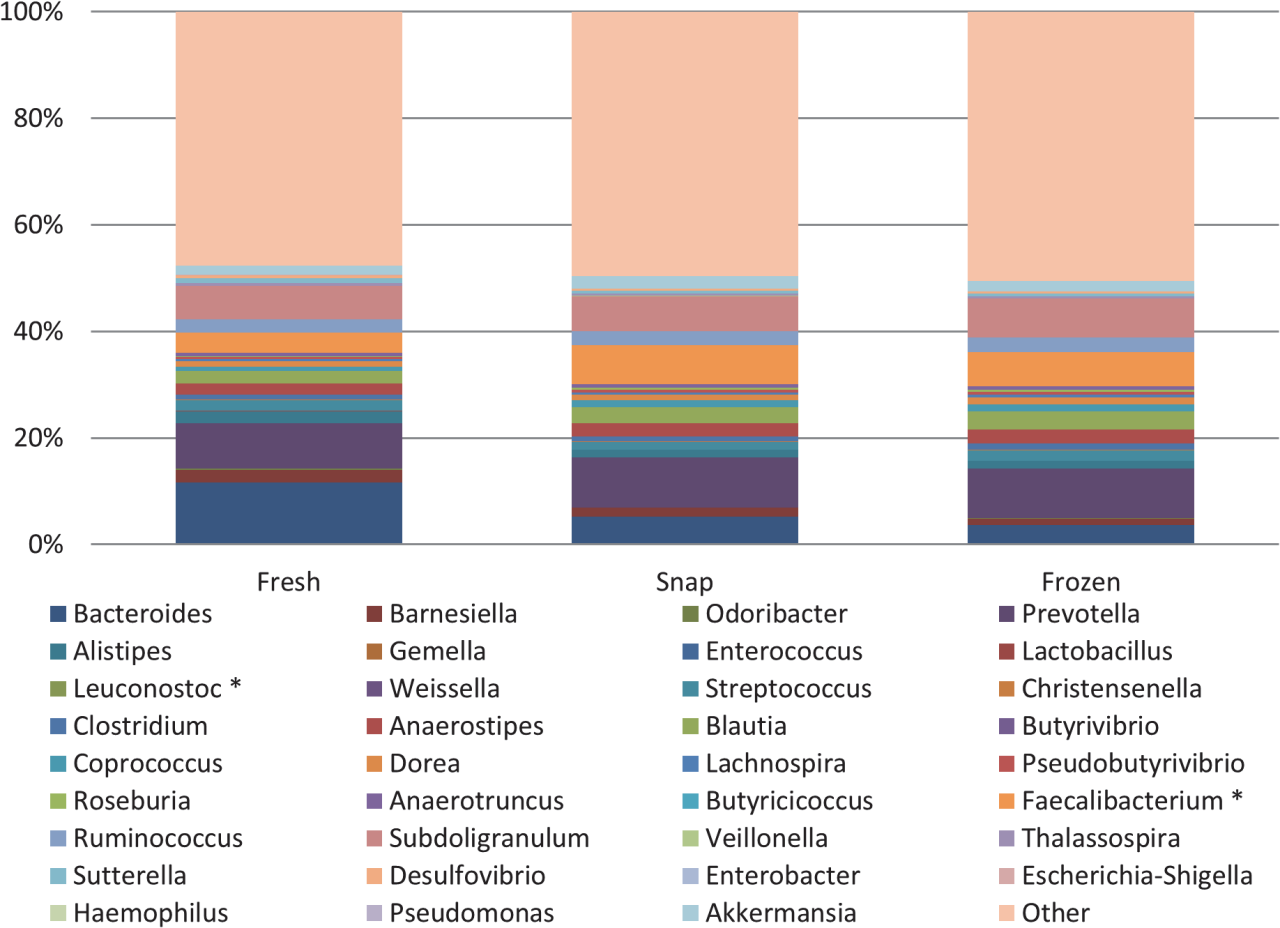
Relative abundances of bacterial genera in the fresh, snap and frozen samples. Statistically significant differences in genera are indicated with an asterisk (*) (p<0.05). The Other category contains all other genera present at <0.01% of assignable reads at genus level. No significant differences in any of these genera between the 3 groups was found using non-parametric Kruskall-Wallis analysis, where statistical significance was accepted as p<0.05, adjusted for ties.

At genus level, again all 3 treatment groups were very similar in the genera that were detected (Fig. 3). All genera detected in fresh samples were detected in the snap and -80°C frozen samples, and at very similar levels, with only 2 significant differences being noted between genera in the different treatment groups. The proportion of *Faecalibacterium* was significantly higher in snap frozen samples compared to fresh samples (p = 0.029). No significant differences were found between proportions of *Faecalibacterium* in the fresh and the -80°C frozen samples (p = 0.055) or between the -80°C frozen samples and snap samples (p = 0.443). In contrast, the proportion of *Leuconostoc* was significantly higher in the fresh samples compared to the snap frozen samples (p = 0.033), but no differences occurred between the fresh and -80°C frozen samples (p = 0.054), or between the snap and -80°C frozen samples (p = 1.000). No significant differences in any of the other genera including proportions of *Bifidobacterium* (p = 0.591), *Bacteroides* (p = 0.667), *Parabacteroides* (p = 0.546), *Pseudomonas* (p = 0.880) or *Clostridium* (p = 0.932) were seen between the 3 groups.

### Diversity analysis

To determine if alterations in microbial diversity occurred as a result of different faecal sample storage conditions, alpha and beta diversity were investigated. No significant difference in alpha diversity occurred between the fresh, snap or -80°C frozen samples, as determined using Chao 1 (p = 0.905), Simpson’s diversity index (p = 0.754) and Shannon index tests (p = 0.662) (Table 1).

**Table 1:** Estimates of alpha diversity for the fresh, −80°C frozen and snap frozen samples.

Data set	Fresh	Frozen −80°C	Snap frozen	p-value
**Chao1 richness estimate**	846	964	878	0.905
**Shannon index**	5.75	5.97	5.87	0.662
**Simpson's diversity index**	0.95	0.96	0.95	0.754
**Number of observed species**	813	927	816	0.865

**Fig 4:**
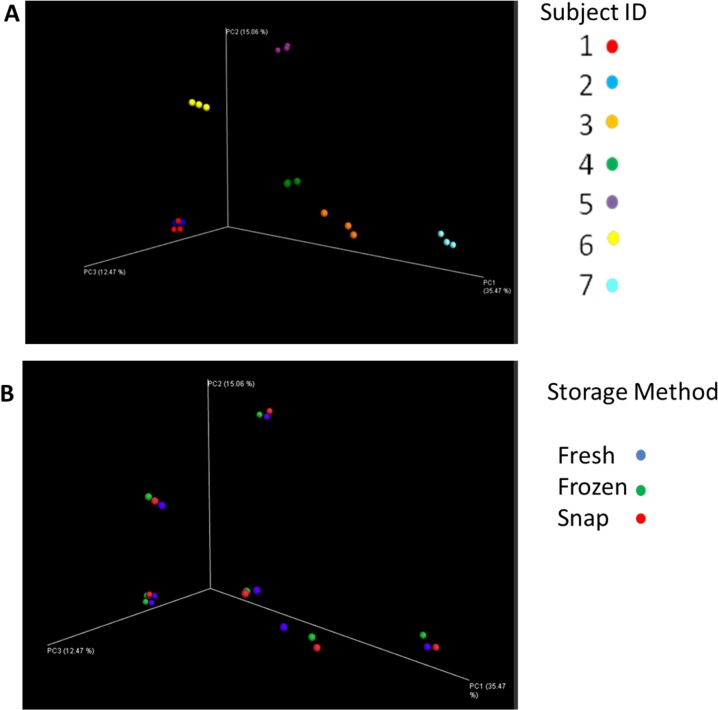
Visualisation of the PCoA analysis based on unweighted UniFrac distance matrices. Results indicate samples separate by subject ID rather than storage method. **Section A**: samples by subject ID. **Section B:** samples by storage method.

To determine if any significant differences in beta diversity occurred based on storage of the samples, PCoA plots were constructed based on weighted and unweighted UniFrac distance matrices. We sought to investigate if samples from the same individual would cluster together. As shown in Fig. 4A, samples clustered according to the individual, with all 3 samples from the one individual clustering more closely together than to other samples from the same storage group (Fig. 4B). The same was also true when the PCoA plots were constructed using weighted UniFrac distance matrices (data not shown).

## Discussion

Despite high-throughput sequencing providing considerable insights into the microbiota of complex environments, including the human gut microbiota, many factors [9, 16], including sample storage prior to DNA extraction, are known to impact on the accuracy of the results achieved. While studies have investigated this, to date however, no study has used MiSeq sequencing to determine the effects of freezing on the faecal microbiota compared to the fresh sample. Aware of the increased sequencing depth and coverage achieved using MiSeq sequencing compared to 454-pyrosequencing, our aim was to determine if subtle changes occur following freezing of faecal samples that may not have been captured in previous studies using 454-pyrosequencing. Such studies are important, as researchers are increasingly using MiSeq sequencing instead of 454-pyrosequencing and thus our study aimed to clarify the most appropriate storage of faecal samples, prior to DNA extraction for MiSeq sequencing to limit biasing the results achieved. While DNA extraction from fresh faecal samples may be the ideal choice, in many situations this is not practical, especially given the growing number of large multicentre projects being undertaken. Thus, knowing the effects of different storage conditions on faecal microbiota, as determined using MiSeq sequencing, is important for the design, analysis and interpretation of microbiota studies in the future.

Our study has further novelty as we investigated the ability to culture total aerobes, anaerobes and bifidobacteria from the snap and -80°C frozen samples compared to the fresh samples. We chose to culture and enumerate total aerobes and anaerobes as the best method of evaluating the global effect of storage condition on the culturable microbiota. Bifidobacteria were cultured and enumerated because of their low abundance in faecal microbiota and their assumed sensitivity to freezing. We rationalised that if bifidobacteria were recoverable following freezing at -80°C, accordingly other low abundance groups may be recoverable. However, the recovery rate of other such groups has yet to be investigated. Although microbiological culturing from faecal samples is for the most part performed on fresh samples [17, 18], there is little information in the literature comparing the effects of storage conditions on the culturable bacterial populations present in faecal samples. Using culturing approaches, our results demonstrated that the levels of total aerobes, total anaerobes or bifidobacteria in the fresh samples were similar compared with either the snap frozen or -80°C frozen samples. The greatest differences were seen between samples from different individuals, rather than from different treatments. The results suggest that rapid freezing of samples following collection and the avoidance of prolonged periods at room temperature or the occurrence of freeze-thaw enables the recovery of comparable levels of aerobes, anaerobes and bifidobacteria from frozen samples, as from fresh samples. Snap freezing did not appear to afford any additional benefits over freezing the samples at -80°C. Importantly, we show that immediate freezing at -80°C demonstrates no significant difference in enumerated total aerobes, total anaerobes and bifidobacteria from fresh or snap frozen, at global level without the addition of glycerol or other preservation media, such as RNA later. We acknowledge the limitations of this study, in that we can only comment on the global levels of the groups enumerated. In future studies, it may be interesting to examine the effects of storage on a wider range of bacterial groups and to investigate if the recovery of bacterial numbers at a global level translates to specific taxa.

When the microbiota of the fresh, frozen and snap frozen samples were compared using MiSeq sequencing, no significant differences occurred at phylum or family levels. The dominant phyla, families and genera were similar between the three groups. We did note non-significant higher levels of *Firmicutes* in the snap and -80°C frozen samples compared to the fresh samples. This supports previous research using qPCR, which demonstrated a significantly higher ratio of *Firmicutes* to *Bacteroidetes* in frozen samples compared to fresh [19]. This may be due to an increased extraction ability of DNA from Gram positive (*Firmicutes*) bacteria following frozen storage [19]. Our results of minimal and non-significant differences in microbiota following freezing of faecal samples are in agreement with previous research which used alternative analysis [11, 20, 21]. The increased depth of coverage achieved through MiSeq enabled us to identify genera present at very low levels e.g. *Bifidobacterium* at <0.001% of assignable phylum reads. However, even in these circumstances no significant difference occurred in samples stored under different storage conditions. Thus, using MiSeq we could confirm the suitability of sample freezing prior to DNA extraction, with results being comparable to fresh samples.

Though our research only investigated short-term (7 days) storage at -80°C, others who have investigated more prolonged storage at -80°C (up to 137 days) have also failed to find any significant effects on faecal microbiota [11, 22]. However, we hope to further study these faecal samples after months and even years at -80°C, using both culture and MiSeq analysis to determine the effects of long-term storage of samples at -80°C on faecal microbiota. The research to date suggests that freezing, either snap or at -80°C, preserves the faecal microbiota, while long-term storage at 4°C, room temperature or samples which undergo freeze-thaw, results in altered microbiota compared to their fresh equivalent [8, 21]. Our fresh samples were processed within 4 hours of collection, during which time they were stored at 4°C. Such a short period of cold storage is unlikely to alter the results of these ‘fresh’ samples compared to the samples when defecated, with previous publications indicating minimal changes occur in faecal microbiota following short-term storage at room temperature or 4°C [11, 20]. In fact, recent studies on sputum samples from individuals with cystic fibrosis have suggested that 12 hours room temperature storage prior to -80°C storage, is unlikely to alter microbiota [23]. Studies examining the effects of snap freezing are limited, but those which have been completed suggest freezing (either on dry ice or at -80°C) achieves the most accurate microbiota relative to the fresh sample [24]. Though snap freezing results in samples reaching the same end-point temperature as samples placed at -80°C, the temperature drops at a greater rate, reducing ice crystal formation in the sample, thus retaining superior cell integrity. Though outside the scope of this study, it may be the case that snap freezing would be beneficial in cases where subsequent enzymatic or RNA-based analysis are to be completed [25]. This study also investigated microbial diversity and found no significant differences between the 3 treatment groups in terms of alpha or beta diversity. Previously, 454-pyrosequencing studies suggested that samples cluster based on the donor rather than the storage of the faecal sample [22]. Our MiSeq results support these findings, with the greatest variation in microbiota being attributable to interpersonal differences rather than the storage treatment.

In conclusion, this is the first MiSeq-based study to examine the effects of different freezing methods on the faecal microbiota compared to fresh samples. Despite the increased sequencing depth achievable using MiSeq as compared to 454-pyrosequencing, our results support previous 454-pyrosequencing results that found the greatest differences between samples is due to the uniqueness of each individual’s microbiota, rather than storage conditions prior to DNA extraction. Our results suggest that rapid freezing, either on dry ice or simply placed at -80°C prior to culturing or DNA extraction for sequencing analysis, will result in accurate microbiota results. Going forward, when comparing data sets from different studies, it will be important to consider how the samples were stored prior to extraction, with results from fresh or frozen samples being more reliable than samples stored at room temperature or 4°C for prolonged periods.
